# Time Trends in Adolescent Diagnoses of Major Depressive Disorder and Co-occurring Psychiatric Conditions in Electronic Health Records

**DOI:** 10.21203/rs.3.rs-4925993/v1

**Published:** 2024-09-18

**Authors:** Marina Wilson, Hyunjoon Lee, Lorenza Dall’Aglio, Xinyun Li, Anushka Kumar, Mary K. Colvin, Jordan W. Smoller, William R. Beardslee, Karmel W. Choi

**Affiliations:** Center for Precision Psychiatry, Department of Psychiatry, Massachusetts General Hospital; Center for Precision Psychiatry, Department of Psychiatry, Massachusetts General Hospital; Center for Precision Psychiatry, Department of Psychiatry, Massachusetts General Hospital; Center for Precision Psychiatry, Department of Psychiatry, Massachusetts General Hospital; Center for Precision Psychiatry, Department of Psychiatry, Massachusetts General Hospital; Department of Psychiatry, Massachusetts General Hospital; Center for Precision Psychiatry, Department of Psychiatry, Massachusetts General Hospital; Department of Psychiatry, Boston Children’s Hospital; Center for Precision Psychiatry, Department of Psychiatry, Massachusetts General Hospital

**Keywords:** adolescence, major depressive disorder, electronic health records, comorbidity, depression, time trends

## Abstract

Major depressive disorder (MDD) is highly prevalent in youth and generally characterized by psychiatric comorbidities. Secular trends in co-occurring diagnoses remain unclear, especially in healthcare settings. Using large-scale electronic health records data from a major U.S. healthcare system, we examined the prevalence of MDD diagnoses and co-occurring psychiatric conditions during adolescence (12–18 years; N = 133,753) across four generations (birth years spanning 1985 to 2002) and by sex. Then using a phenome-wide association analysis, we explored which of 67 psychiatric conditions were associated with adolescent MDD diagnosis in earlier versus recent generations. Adolescent MDD diagnosis prevalence increased (8.9 to 11.4%) over time. Over 60% with an MDD diagnosis had co-occurring psychiatric diagnoses, especially neurodevelopmental and anxiety disorders. Co-occurring diagnoses generally increased over time, especially for anxiety disorders (14 to 50%) and suicidal behaviors (6 to 23%), across both sexes. Eight comorbidities interacted with generation, showing stronger associations with MDD diagnosis in earlier (e.g., conduct disorder) versus more recent (e.g., suicidal ideation and behaviors) generations. The findings underscore the importance of assessing psychiatric complexity in adolescents diagnosed with MDD, applying transdiagnostic approaches to address co-occurring presentations, and further investigating potential causes for generational increases.

## Introduction

Major depressive disorder (MDD) is a debilitating disorder characterized by persistently low or depressed mood, loss of pleasure, and associated cognitive and somatic symptoms (American Psychiatric Association, 2022). Although MDD onsets throughout the life course, one in five youth in the United States (US) will experience MDD before 18 years, with data suggesting nearly half of youth experiencing depression will actually be diagnosed before adulthood (Costello et al., 2002; Goodwin et al., 2022; Kessler et al., 2001; Lewinsohn et al., 2000). Adolescent-onset MDD is associated with negative long-term outcomes, including chronic depression through adulthood, disrupted employment, and reduced educational attainment (Clayborne et al., 2019; Johnson et al., 2018; Thapar et al., 2022).

In youth with MDD, up to 90% have psychiatric comorbidities (Birmaher et al., 2007). MDD may develop following other psychiatric conditions (e.g., anxiety, ADHD) or may be associated with increased risk of developing others (e.g., conduct and substance use disorders) (Lewinsohn et al., 1998; Rohde et al., 1991). MDD-related psychiatric comorbidity in youth has been linked to adverse outcomes (e.g., health concerns, incarceration, high school dropout, etc.) (Copeland et al., 2015; Zimmerman & Mattia, 1999), and poor treatment response (Weisz et al., 2017). This highlights the importance of accounting for psychiatric comorbidities for adolescent MDD during the patient’s clinical presentation and treatment.

To date, studies examining comorbidities (e.g., anxiety disorder, substance use, etc.) of adolescent depression have been based on clinical, epidemiological, or community-based samples where individuals are selected based on having the disorder and being treated for the disorder (e.g., recruited from a psychiatry clinic) (Angold & Costello, 1993; Avenevoli et al., 2015; Kovacs et al., 1989). These studies have identified that youth with depression have a high likelihood of having other psychiatric disorders (Angold & Costello, 1993; Avenevoli et al., 2015). Yet, the prevalence and comorbidity rates of adolescent MDD diagnoses in healthcare settings, which may include psychiatry clinics but also broader settings, are largely unexplored, which may limit the ability of clinicians and systems to anticipate such complex presentations. Electronic health records (EHR) from healthcare settings could offer novel insights into the diverse psychiatric diagnoses that co-occur with adolescent MDD. EHRs not only permit the examination of hundreds of thousands of individuals, lending greater power than prior studies, but also capture clinically recognized presentations of adolescent depression in real-world settings. Although several previous studies have utilized EHRs to examine general childhood mental health disorders, adolescent attention-deficit/hyperactivity disorder (ADHD), or substance use disorders along with their psychiatric comorbidities (Elia et al., 2023; Slaby et al., 2022; Wu et al., 2011), they have not focused on MDD, one of the most common and impairing conditions in youth.

Moreover, less is known about secular trends of adolescent MDD and co-occurring psychiatric diagnoses in healthcare settings examining adolescents passing through the health system in different generations (e.g., in the early 2000s versus 2010s). It has been reported that MDD, and comorbid psychiatric conditions like ADHD (Davidovitch et al., 2017), have each increased over time in young people (Keyes et al., 2019; Mojtabai et al., 2016). Changes over time in EHR-based prevalence of MDD and co-occurring psychiatric diagnoses in youth could give us insight into evolving clinician diagnostic practices, may reflect rising societal awareness and help-seeking for these conditions, or index real increases in the incidence of these mental health conditions alongside MDD. If comorbid diagnoses are becoming more common, treatment and care services must be tailored accordingly. However, how diagnostic patterns for adolescent-onset MDD and potentially co-occurring psychiatric conditions have changed across generations has been largely unexplored with systematically recorded hospital data.

Leveraging over 20 years of data from a large EHR-based healthcare system in the Boston area, we aimed to (1) describe the proportion of individuals with an adolescent MDD diagnosis who also received diagnoses during adolescence in other major psychiatric categories (e.g., anxiety disorders, substance use disorders), and to examine these patterns over time stratifying by generation and sex, and (2) identify which specific psychiatric conditions are associated with an adolescent MDD diagnosis in more recent versus earlier generations. Such findings could inform diagnostic conceptualization and treatment/service planning.

## Methods

The Strengthening the Reporting of Observational studies in Epidemiology (STROBE) cohort checklist (**Supplementary File 1**) was used when writing this report (von Elm et al., 2014).

### Data source and study population

**Figure 1:**
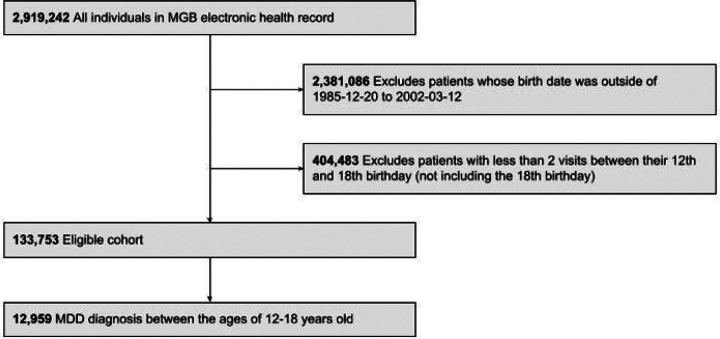
Flow Diagram of Study Sample

Data for this study were drawn from the Mass General Brigham (MGB) Research Patient Data Registry (Nalichowski et al., 2006), an EHR database that spans 23 years of data from more than 6.5 million patients treated at Massachusetts General Hospital, Brigham and Women’s Hospital, and affiliated community/specialty hospitals in the Boston area for a retrospective cohort study. Secondary analysis of these deidentified data was approved by the MGB Institutional Review Board. Our study sample included patients with at least two EHR-documented visits on separate dates during their adolescent window, defined as between ages 12 (inclusive) to 18 (non-inclusive) (n = 133,753). Age 12 was chosen based on when children transition to adolescent and young adult medicine in our system, and age 18 was chosen based on the legal age of majority. In this sample (Fig. 1), 12,959 (9.7%) patients had received at least one International Classification of Diseases (ICD; ninth and tenth editions) code for MDD during this adolescent time window with the list of possible codes reported in **Supplementary Table 1**.

#### Descriptive Analyses

##### Prevalence of adolescent MDD diagnosis

We first examined the prevalence of adolescent MDD diagnosis in the overall sample and when stratified by sex (male vs. female) and birth year group (1985–1989, 1990–1994, 1995–1999, and 2000–2002). For these four birth year groups, the corresponding adolescent windows (i.e., when individuals would encounter the health system between ages 12 to 18) are: 1997–2007, 2002–2012, 2007–2017, and 2012–2020. Because the earliest recorded visit in our EHR data was 12/20/1997, we limited the earliest birth year group to patients born on or after 12/20/1985 to ensure every patient had the same potential observation window starting from their 12th birthday. Similarly, in the most recent birth year group (2000–2002), we censored the latest birth date at 3/12/2002 to ensure every patient had the same potential observation window until their 18th birthday (non-inclusive) and prior to the COVID-19 pandemic lockdown (3/13/2020). Using chi-square tests, we examined whether the prevalence of adolescent MDD diagnosis was significantly different between male and female patients, and between each successive pair of birth year groups (1985–1989 vs. 1990–1994, 1990–1994 vs. 1995–1999, and 1995–1999 vs. 2000–2002).

##### Prevalence of co-occurring diagnoses

For this study, we will be using the phrase “co-occurring diagnoses” to reflect diagnoses that are received during the same adolescent window (regardless of temporal precedence) rather than speculating on underlying comorbidity in general. Among those with an adolescent MDD diagnosis (n = 12,959), we examined co-occurring diagnoses from five psychiatric conditions categories: (1) anxiety disorders; (2) substance use disorders (SUDs); (3) suicidal ideation and behaviors; (4) severe mental disorders (i.e., bipolar disorder, schizophrenia, and psychosis); and (5) neurodevelopmental disorders (i.e., attention deficit-hyperactivity disorder (ADHD), autism spectrum disorder (ASD), and learning disabilities). The list of diagnoses that fall within these categories can be found in **Supplementary Table 2**. These categories were informed by literature and domain knowledge on commonly co-occurring conditions in youth with MDD (Angold & Costello, 1993; Cameron, 2007; Cumyn et al., 2009; Kovacs et al., 1989; Swendsen & Merikangas, 2000). As above, we analyzed the prevalence of at least one co-occurring diagnosis (based on having at least one related ICD code between ages 12 and 18) in each of the five psychiatric categories, overall and stratified by sex and birth year group. Chi-square tests were conducted to determine sex and generational differences (between successive pairs of birth year groups). Additionally, we characterized the number of co-occurring psychiatric disorder categories among those with an adolescent MDD diagnosis (i.e., “multimorbidity”), stratified by sex and birth year group.

Although we focused on individuals who had received any adolescent MDD diagnosis (1 + ICD codes), as a sensitivity analysis, we also assessed whether descriptive patterns remained consistent using a slightly more stringent threshold to increase the likelihood of a valid signal (2 + ICD codes).

##### Generation-interaction PheWAS Analyses

Next, we took a more granular and data-driven approach to identify psychiatric conditions in which the association with EHR-based adolescent MDD diagnosis differed across generations. To achieve this, we conducted a psychiatric phenome-wide association study (PheWAS) (Denny et al., 2010) including a generation*adolescent MDD diagnosis interaction term in each logistic regression: for simplicity, we stratified the sample into two groups reflecting early (birth year 1985–1994; N = 17,716) and more recent (birth year 1995–2002, N = 25,619) generations.

First, we constructed the psychiatric phenome by extracting all psychiatric conditions via ICD-9 and ICD-10 codes mapped to 73 umbrella phenotype codes (“phecodes”) using the *PheWAS* R package (Carroll et al., 2014) between the patients’ 12th (inclusive) and 18th (non-inclusive) birthdays, which provides a more expansive set of phenotypes than the targeted conditions in the earlier analyses. For each psychiatric phecode (outcome, coded as present/absent based on having at least two ICD codes during the adolescent window), we fit a logistic regression model using adolescent MDD diagnosis (exposure) and generation*adolescent MDD diagnosis interaction term, adjusting for sex, race, ethnicity, and healthcare utilization, indexed by ICD code count during the adolescent window. Associations were computed only for phecodes recorded in at least 10 patients. We identified phecodes in which generation*MDD diagnosis interaction term was below the Bonferroni-adjusted p-value (7.8 × 10^− 4^).

## Results

### Descriptive Analysis

#### Sample characteristics

Sample characteristics are shown in **Supplementary Table 3**. Our sample was approximately half female (52.3%) and predominantly White (68.8%), non-Hispanic (94.8%), and born between 1995 and 2002 (56.5%). Compared to the overall study population, adolescent MDD patients had a higher proportion of females (62.6%) and individuals born in the most recent generation (61.6%).

#### Prevalence of adolescent MDD diagnosis

The prevalence of adolescent MDD diagnosis in our study population, overall as well as stratified by sex and birth year group, is reported in Table 1. Adolescent MDD diagnosis (9.7% overall) was more prevalent among female than male patients (12% female vs. 8% male, χ^2^ = 609.3, *p* < 2.2e^− 16^). The prevalence of adolescent MDD diagnosis was in the 8–9% range in the earliest two birth year groups, and increased to 10–11% range in the more recent birth year groups. The overall prevalence of adolescent MDD diagnosis was lower (6.4%) using a more stringent definition (2 + instead of 1 + ICD codes), but the patterns noted above remained consistent as reported in **Supplementary Tables 4 and 5**.

#### Prevalence of co-occurring psychiatric disorders

**Figure 2:**
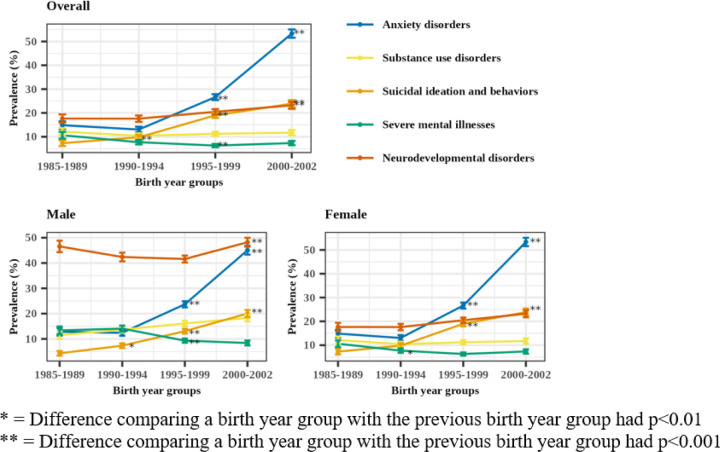
Prevalence of co-occurring diagnosis in each major psychiatric category among adolescent MDD patients across birth year groups.

The prevalence of a co-occurring diagnosis in each of the five major psychiatric categories among adolescent MDD patients, overall and stratified by sex and birth year group, is reported in Table 1 and Fig. 2. The prevalence (base rates) of these diagnoses in the overall adolescent sample is provided in **Supplementary Table 6**. Approximately 60% of adolescents with MDD had at least one co-occurring diagnosis in the five psychiatric disorder categories (67% male and 55% female). Patterns were consistent in sensitivity analyses using a more stringent definition of adolescent MDD diagnosis as reported in **Supplementary Table 7**.

#### Anxiety disorders

Approximately 27% of adolescent MDD patients had a co-occurring anxiety disorder diagnosis between the ages of 12 and 18 (Table 1). Co-occurring anxiety disorder diagnosis was more prevalent among female than male patients (28% female vs. 24% male, *p* = 5.3e^− 07^). As shown in Fig. 2, the prevalence of co-occurring anxiety disorder diagnosis ranged from 12–14% in the earliest two birth year groups, doubled to 26% in the next birth year group (*p* < 2.2e^− 16^) and further doubled to 50% in the last birth year group (*p* < 2.2e^− 16^).

#### Substance use disorders (SUDs)

Overall, 13% of adolescent MDD patients had a co-occurring SUDs diagnosis between the ages of 12 and 18 (Table 1). Co-occurring SUDs diagnosis was more prevalent among males than females (15% male vs. 11% female, *p* = 3.3e^− 11^). As shown in Fig. 2, the overall co-occurrence of MDD with SUDs diagnoses stayed relatively constant over the birth year groups (ranging from 12–14%), although there was a qualitative difference between the earliest and last birth year group among males (11 vs 18%).

#### Suicidal Ideation and Behaviors

Overall, 15% of adolescent MDD patients had a co-occurring code for suicidal ideation and behaviors between the ages of 12 and 18 (Table 1). Co-occurring code for suicidal ideation and behaviors was more prevalent among female than male patients (16% female vs. 12% male, *p* = 6.2e^− 12^). As shown in Fig. 2, the prevalence of co-occurring code for suicidal ideation and behaviors increased from 6–9% (*p* = 0.0006) between the earliest two birth year groups, nearly doubled to 17% in the next birth year group (p < 2.2e^− 16^) and increased to 23% in the last birth year group (*p* = 4.2e^− 10^).

#### Severe mental illness (SMI)

Overall, 9% of adolescent MDD patients had a co-occurring SMI diagnosis between the ages of 12 and 18 (Table 1). Co-occurring SMI diagnosis was more prevalent among male than female patients (11% male vs. 8% female, *p* = 3.5e-11). As shown in Fig. 2, the prevalence of co-occurring SMI diagnosis ranged from 10–12% in the earliest two birth year groups, significantly decreased to 7.4% in the next birth year group (*p* = 2.0e-05) and remained constant at 7.7% in the last birth year group.

#### Neurodevelopmental disorders

Overall, 29% of adolescent MDD patients had a co-occurring neurodevelopmental disorder diagnosis between the ages of 12 and 18 (Table 1). Co-occurring neurodevelopmental disorders diagnosis were more prevalent among male than female patients (44% male vs. 20% female, *p* < 2.2e^− 16^). As shown in Fig. 2, the prevalence of co-occurring neurodevelopmental disorders diagnosis ranged from 27–29% in the earliest three birth year groups, then increased to 32% in the last birth year group *(p* = 7.7e^− 05^). Notably, male adolescent MDD patients consistently showed a high prevalence of co-occurring neurodevelopmental disorder diagnosis across all birth year groups, with a higher prevalence than any other psychiatric category across time.

#### Psychiatric multimorbidity

**Figure 3:**
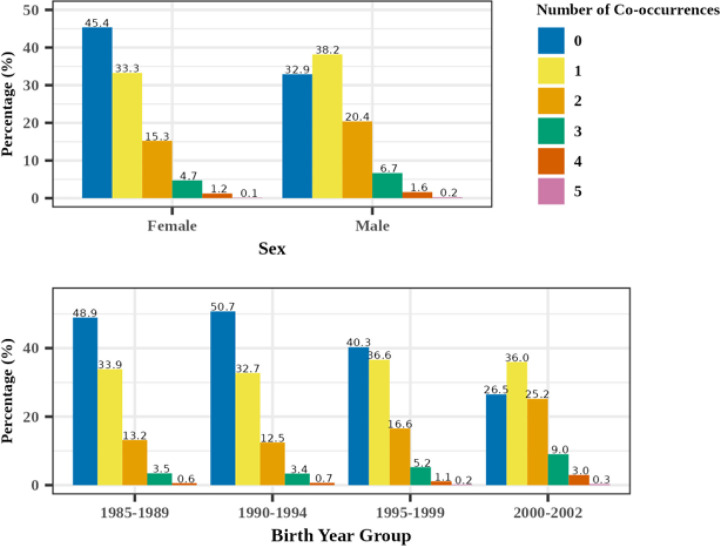
Number of psychiatric categories co-occurring with adolescent MDD diagnosis, stratified by sex and birth year groups.

Patterns of psychiatric multimorbidity are reported in Fig. 3, stratified by sex and birth year group. Multimorbidity, defined here as adolescents with MDD having two or more diagnoses in other psychiatric disorder categories, was higher among male than female patients (29% male vs. 21% female, p < 2.2e^− 16^) and consistently increased with more recent birth year groups (17% for 1985–1989 and 1990–1994, 23% for 1995–1999, 38% for the 2000–2002 birth year group).

#### Moderating effect of generation*adolescent MDD diagnosis on psychiatric phecodes

**Figure 4:**
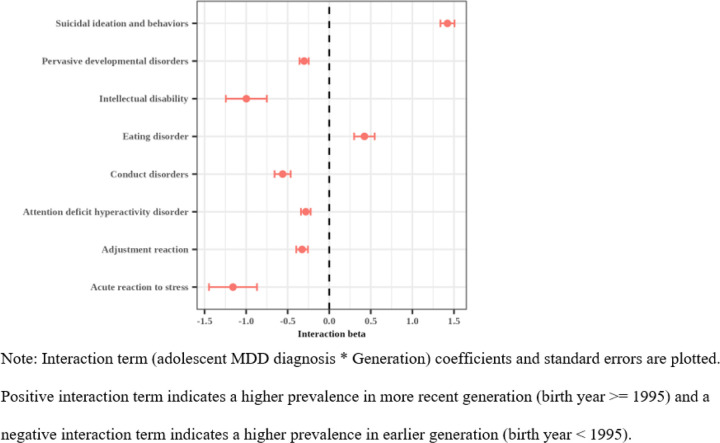
Psychiatric phecodes with significant generation interaction with adolescent MDD diagnosis

Adolescent MDD diagnosis was significantly associated with a greater number of psychiatric disorder phecodes in the more recent generations (k = 32) than the earlier generations (k = 25), as reported in **Supplementary Table 8**. Eight (12.5%) of 64 psychiatric disorder phecodes had a signi cant generation interaction with adolescent MDD diagnosis and are shown in Fig. 4. Six phecodes (acute reaction to stress (B=−1.2, SE = 0.3, p = 6.1E-05); adjustment reaction (B=−0.3, SE = 0.07, p = 2.8E-06); conduct disorders (B=−0.6, SE = 0.1, p = 8.6E-09); pervasive developmental disorders (B=−0.3, SE = 0.06, p = 4.7E08); ADHD (B=−0.3, SE = 0.06, p = 1.4E-06); intellectual disability (B=−1.0, SE = 0.2, p = 5.1E-05)) had stronger associations with adolescent MDD diagnosis in the earlier generation; two phecodes (suicidal ideation and attempt (B = 1.4, SE = 0.08, p = 3.9E-63); eating disorder (B = 0.4, SE = 0.1, p = 6.1E-04)) had stronger associations with adolescent MDD diagnosis in the more recent generation.

## Discussion

In this EHR-based study, we assessed secular time trends in the prevalence of MDD diagnosis and co-occurring diagnostic psychiatric conditions among adolescents in a major general hospital system in the US (*N* = 133,753). Four key findings emerged. First, the prevalence of adolescent MDD diagnosis increased over time, affecting more than one in ten patients between ages 12 and 18. Second, diagnostic comorbidity was the norm for adolescent MDD, with more than 60% of patients receiving another psychiatric diagnosis during adolescence, most frequently neurodevelopmental or anxiety disorders. Third, co-occurring psychiatric diagnoses in patients with adolescent MDD increased over time, with more than three-fold rates of co-occurring diagnoses of anxiety disorders and codes for suicidal ideation and behaviors. Lastly, adolescent MDD diagnosis was significantly associated with a greater number of psychiatric conditions in more recent generations compared to earlier generations, with stronger association of some conditions (e.g., eating disorder) and reduced association of others (e.g., conduct disorder, ADHD) with MDD over time.

Overall, we observed a 9% prevalence rate of adolescent MDD diagnosis in our health system, slightly higher than the 5–7% rate seen in population-based studies (Kessler et al., 2005) or community/clinical samples (Costello et al., 2006). Epidemiological studies may not capture the most severe cases due to study participation and retention challenges. Moreover, our sample is potentially enriched for individuals with higher risk for psychiatric problems, given the hospital-based setting. From 1985–1989 to 2000–2002, the prevalence of adolescent MDD diagnoses in our system increased from 9–11%. Although our data does not allow us to identify the causes of this secular increase, several factors may have contributed. First, epidemiological trends indicate that more youth today are developing depression (Thapar et al., 2022). Second, stigma-reducing interventions for mental health over the past two decades (Walsh & Foster, 2020) (Pescosolido et al., 2021) have likely led to greater care-seeking and diagnosis during this observation period. Third, an increase in adolescent MDD diagnoses may have resulted from increased surveillance, e.g. school-based screenings for mental health issues among youth (Hogan, 2003; National Research Council and Institute of Medicine, 2009; US Department of Health and Human Services et al., 2000).

Among individuals with adolescent MDD, the most common co-occurring diagnostic major psychiatric categories were neurodevelopmental disorders (29%), followed by anxiety disorders (27%), suicidal ideation and behavior (15%), substance use disorders (13%), and severe mental illness (9%) (Table 1) which is consistent with the literature (Angold et al., 1999; Leone et al., 2021; Rosenberg et al., 2011; Sørensen et al., 2005; Weersing et al., 2008). While studies have shown that SMI and MDD are also highly comorbid across the lifetime (32–41%), SMIs typically develop later, in young adulthood (Etchecopar-Etchart et al., 2021; Fusar-Poli et al., 2012; Wilson et al., 2020), which may explain the relatively low co-occurrence in our sample.

As for temporal trends, all major psychiatric categories showed increases in diagnostic co-occurrence with adolescent MDD diagnosis, except for severe mental illness. The distribution of diagnostic co-occurring adolescent psychiatric conditions also shifted over time, with a decrease in the proportion of patients with zero co-occurring diagnoses and an increase in those with at least one or more co-occurring diagnoses, which became more pronounced with each successive birth year group (Fig. 2). Notably, both anxiety- and suicide-related diagnoses tripled from 14–50% and 6–23%, respectively, over time. Prior studies have demonstrated that MDD is a primary risk for suicidal ideation and behaviors (Esposito & Clum, 2002; Kovacs et al., 1993; Nock et al., 2013), yet it is especially concerning that this secular increase has increased in recent generation groups. Importantly, such increase may also reflect more individuals being evaluated and given a diagnosis of MDD in the context of hospital admissions for suicidal behavior. It is also possible that an increase in technology use is influencing the severity of depression in adolescents resulting in an increase in concomitant suicidal ideation and behavior (Twenge, 2020). Increased attention to the links between adolescent MDD, anxiety, and suicidality in healthcare settings is essential.

Compared to anxiety- and suicide-related diagnoses, the diagnostic co-occurrence of substance use and neurodevelopmental disorders with adolescent MDD showed modest increases over time (Table 1). However, males showed an increase in co-occurring substance use disorder diagnoses, and a high prevalence of co-occurring neurodevelopmental disorders diagnoses across all time points relative to other psychiatric categories (Fig. 1), which builds on prior literature (Hunt et al., 2020; Merikangas et al., 2010). The modest increase in diagnostic co-occurring neurodevelopmental disorders might reflect diagnostic changes in ASD, which became more inclusive of milder presentations, and the elimination of the Pervasive Developmental Disorder category, allowing more children to meet an ASD diagnosis (Faroy et al., 2016).

Regarding sex differences, the most commonly diagnostic co-occurring psychiatric categories for adolescent MDD in males were substance use disorders, severe mental illnesses, and neurodevelopmental disorders, versus anxiety disorders and suicidal ideation and behaviors in females. This is consistent with studies suggesting that females are more likely to develop internalizing conditions compared to males (Merikangas et al., 2010; Rescorla et al., 2007). Additionally, males with adolescent MDD showed higher rates of diagnosis with another psychiatric disorder (12.5% higher prevalence) and greater multimorbidity (i.e., two or more comorbidities; 7.5% higher prevalence). Reasons for this remain unclear, warranting further research. While males may have more complex psychiatric presentations (Kovess-Masfety et al., 2021), higher healthcare contact related to certain psychiatric conditions more typically shown in males (e.g., SUDs) may also result in more opportunities to receive other diagnoses, leading to greater complexity in EHR-documented presentations (Chen et al., 2013).

In our PheWAS, we identified eight psychiatric conditions whose association with adolescent MDD were differentially enriched by the generation. Six conditions, including ADHD, conduct disorder, pervasive developmental disorders, and adjustment reaction, were more strongly associated with adolescent MDD in the earlier generations. The reduced co-occurrence of diagnoses of ADHD and MDD over time is somewhat surprising as recent studies have suggested a secular increase in ADHD diagnoses (Davidovitch et al., 2017; Getahun et al., 2013). One possible explanation could be changes in diagnostic systems. For example, with the transition from ICD-9 to ICD-10, which occurred in 2015, the number of available billing codes related to ADHD decreased (e.g., 8 to 6), potentially providing fewer coding opportunities. This was especially marked for conditions like conduct disorder, where the number of billing codes dropped from 23 to 7.

On the other hand, two conditions, speci cally eating disorders and suicidal ideation and behaviors, were more strongly associated with adolescent MDD in the later generations. Incidentally, the available billing codes for these two conditions expanded in the transition from ICD-9 to ICD-10 (7 to 12 and 20 to 25, respectively). A change in the Diagnostic and Statistical Manual of Mental Disorders (e.g., DSM-IV to DSM-V), occurring around 2013, may also explain increased rates of eating disorder diagnoses among adolescents diagnosed with MDD. Specifically, it has been noted that DSM-IV criteria for eating disorders may not have been as applicable to adolescents as it required amenorrhea, which could have excluded various populations (e.g., males, females on birth control) and did not distinguish between overeating and recurrent binge eating, whereas DSM-V removed amenorrhea and added binge eating disorder (American Psychiatric Association, 2022; Nicholls et al., 2000), potentially expanding diagnostic opportunities for young people. The increased association with diagnoses of suicidal ideation and behavior is consistent with what we observed in our earlier analyses, as discussed above. Future work should include a deeper examination of these specific psychiatric conditions that are increasingly co-occurring with adolescent MDD, what might be driving these diagnostic patterns, and the implications they have for treatment. Overall, these findings suggest differential generational patterns of diagnosis of these disorders in the adolescent window when the individual has a MDD diagnosis.

### Limitations

Our analysis of large-scale, real-world health system data allowed us to capture naturalistic patterns of diagnosis over time and across domains of psychopathology. However, our results should be interpreted considering several limitations. First, individuals in our sample may have received MDD or other psychiatric diagnoses outside of the MGB health system, resulting in missing diagnostic outcomes. Second, depression in youth tends to go undiagnosed and untreated, with only half being diagnosed before adulthood (Zuckerbrot et al., 2018). As such, we are only capturing trends in diagnosis in a health system, rather than the natural epidemiology of depression and its comorbidities in the population. Third, our data was gathered from a single regional health system and results may not generalize to other populations (e.g., rural areas, other countries, universal healthcare settings). Fourth, given the hospital-based setting Berkson’s bias may be inflating the correlation between MDD and other diagnoses because the sample is not drawn from a general population. Moreover, we defined adolescent MDD using 1 + ICD codes, which may be noisy and have low positive predictive value for true major depressive disorder. However, similar results were found when using 2 + ICD codes for MDD case Classification (6.4%) (**Supplementary Tables 5 and 7**), and precisely identifying true MDD cases was beyond the scope of this work. Fifth, the window chosen for adolescence is somewhat arbitrary window as there is heterogeneity in the age ranges used to de ne adolescence (Sawyer et al., 2018). Finally, the temporal ordering of such co-occurring psychiatric diagnoses and age of youth was not investigated and could be tackled by future research.

## Conclusion

Our study in a major healthcare system spanning 133,753 patients over 23 years reveals temporal trends in adolescent MDD and its diagnostic co-occurrence with other psychiatric conditions. Fifindings highlight a growing prevalence of MDD diagnosis among adolescent patients and concurrent rise in the prevalence of co-occurring psychiatric diagnoses, particularly for anxiety disorders and suicidal behavior, suggesting the increasing importance of addressing multiple conditions. While further work is needed, shifts in how healthcare providers approach the assessment and diagnosis of these disorders may have contributed to observed trends. Continued research on the influence of insurance policies, cultural shifts in mental health stigma, education programs, and ever-changing diagnostic criteria for psychiatric disorders on assessment and treatment is needed. Regardless, findings point to the importance of carefully assessing comorbidities in adolescents with MDD in both clinical and research settings and tailoring therapeutic approaches for complex phenotypic presentations, with perhaps more emphasis on utilizing a transdiagnostic approach. Further research is needed to explore these trends and develop more effective prevention and treatment strategies for mental health conditions that increasingly co-occur in adolescents.

## Figures and Tables

**Table 1:** Prevalence of adolescent MDD diagnosis in study population and co-occurring diagnoses among adolescent MDD patients

Disorder	Birth Year Group	All (%)	Male (%)	Female (%)
MDD	1985–1989	8.9 [8.5–9.3]	7.8 [7.2–8.3]	9.9 [9.4–10.5]
	1990–1994	8.5 [8.2–8.7]	6.8 [6.4–7.2]*	10.0 [9.5–10.4]
	1995–1999	10.0 [9.7–10.2]**	7.5 [7.2–7.9]*	12.3 [11.9–12.7]**
	2000–2002	11.4 [11.1–11.8]**	8.7 [8.2–9.2]**	14.0 [13.4–14.5]**
	Total	9.7 [9.5–9.8]	7.6 [7.4–7.8]†	11.6 [11.4–11.8]
Co-occurring Anxiety Disorders	1985–1989	14.0 [13.6–14.5]	12.8 [10.4–15.2]	14.9 [12.8–16.9]
	1990–1994	12.8 [12.5–13.1]	12.4 [10.5–14.3]	13.0 [11.5–14.5]
	1995–1999	25.6 [25.2–26.0]**	23.7 [21.7–25.7]**	26.6 [25.1–28.2]**
	2000–2002	50.3 [49.7–50.9]**	45.1 [42.2–47.9]**	53.3 [51.1–55.5]**
	Overall	26.8 [26.0–27.6]	24.3 [23.1–25.5]†	28.3 [27.3–29.3]
Co-occurring Substance Use Disorders (SUDs)	1985–1989	11.9 [11.4–12.3]	11.5 [9.2–13.8]	12.2 [10.2–14.1]
	1990–1994	11.6 [11.3–12.0]	13.7 [11.7–15.6]	10.4 [9.0–11.8]
	1995–1999	13.0 [12.7–13.3]	16.1 [14.4–17.9]	11.2 [10.1–12.3]
	2000–2002	14.1 [13.7–14.5]	18.3 [16.0–20.5]	11.7 [10.3–13.1]
	Overall	12.8 [12.2–13.4]	15.3 [14.3–16.3]†	11.3 [10.6–12.0]
Co-occurring Suicidal Ideation and Behaviors	1985–1989	6.1 [5.8–6.4]	4.3 [2.9–5.8]	7.3 [5.8–8.8]
	1990–1994	8.8 [8.5–9.1]**	7.3 [5.9–8.8]*	9.8 [8.5–11.1]
	1995–1999	16.8 [16.5–17.2]**	13.1 [11.5–14.6]**	19.0 [17.6–20.4]**
	2000–2002	22.5 [22.0–23.0]**	20.0 [17.7–22.4]**	23.9 [22.0–25.8]**
	Overall	14.7 [14.1–15.3]	12.0 [11.0–12.9]†	16.4 [15.6–17.2]
Co-occurring Severe Mental Illnesses (SMIs)	1985–1989	11.7 [11.3–12.2]	13.4 [10.9–15.8]	10.6 [8.8–12.4]
	1990–1994	10.2 [9.8–10.5]	14.1 [12.1–16.0]	7.7 [6.5–8.9]*
	1995–1999	7.4 [7.2–7.6]**	9.3 [8.0–10.7]**	6.3 [5.4–7.2]
	2000–2002	7.7 [7.4–8.0]	8.4 [6.8–10.0]	7.3 [6.2–8.5]
	Overall	8.8 [8.3–9.2]	10.9 [10.0–11.8]†	7.5 [6.9–8.1]
Co-occurring Neurodevelopmental Disorders	1985–1989	29.2 [28.6–29.8]	46.6 [43.0–50.2]	17.6 [15.4–19.9]
	1990–1994	27.1 [26.6–27.5]	42.4 [39.6–45.2]	17.6 [15.9–19.3]
	1995–1999	28.2 [27.7–28.5]	41.6 [39.3–43.9]	20.5 [19.0–21.9]
	2000–2002	32.3 [31.8–32.9]**	48.2 [45.3–51.1]**	23.2 [21.3–25.0]
	Overall	29.1 [28.2–29.8]	44.1 [42.7–45.5]†	20.1 [19.2–20.9]

* = Difference comparing a birth year group with the previous birth year group had p < 0.01

** = Difference comparing a birth year group with the previous birth year group had p < 0.001

† = Difference between male and female had p < 0.001
